# ADAMTS2: More than a procollagen N-proteinase

**DOI:** 10.1016/j.gendis.2025.101686

**Published:** 2025-05-13

**Authors:** Ruben Vanlerberghe, Alain Colige, Anne-Marie Malfait, Delfien Syx, Fransiska Malfait

**Affiliations:** a Center for Medical Genetics, Ghent University Hospital, Department of Biomolecular Medicine, Ghent University, Ghent 9000, Belgium; b Laboratory of Connective Tissues Biology, Tour de Pathologie, GIGA-Cancer, University of Liège, Liège 4000, Belgium; c Division of Rheumatology, Department of Internal Medicine, Rush University Medical College, and Chicago Center on Musculoskeletal Pain, Chicago, IL 60612, United States

**Keywords:** ADAMTS2, Collagen, Dermatosparaxis, Ehlers-Danlos syndrome, Procollagen N-Proteinase

## Abstract

A disintegrin and metalloproteinase with thrombospondin motifs 2 (ADAMTS2) is a member of the ADAMTS zinc metalloproteinase family, best known for its role as a procollagen I N-proteinase in the maturation of fibrillar collagens. Biallelic defects in the *ADAMTS2* gene*,* resulting in a loss of ADAMTS2 enzyme activity and consequent retention of N-propeptides in type I procollagen molecules, lead to the rare monogenic disease Ehlers-Danlos syndrome dermatosparaxis type (dEDS) in humans, and dermatosparaxis in animals, conditions that are hallmarked by extreme fragility of the skin and other soft connective tissues. Recent studies have expanded the substrate repertoire of ADAMTS2 considerably, revealing its potential implication in several biological processes, including angiogenesis, lymphangiogenesis, neurodevelopment, immunity, and spermatogenesis. There is also emerging evidence for a role for ADAMTS2 in complex disorders, including cancer and cardiovascular and neurodegenerative disease. These findings may not only provide answers to hitherto unsolved questions in dermatosparaxis but also unveil a therapeutic and/or biomarker potential of ADAMTS2 in many diseases. This narrative review provides an in-depth overview of the discovery, structure, regulation, and enzymatic role of ADAMTS2, its role in fibrillar collagen maturation and in dEDS pathogenesis, as well as its newly discovered substrates and its potential role in complex disorders.

## The discovery of a type I procollagen N-proteinase

Collagens are the most abundant proteins in humans, accounting for approximately one-third of all protein content.^1^ As the most prevalent extracellular matrix (ECM) component, collagens provide structural strength and integrity to many tissues.^2^ The 28 members of the collagen superfamily (encoded by 44 different genes^3^) take part in diverse biological processes, including tissue repair and cell adhesion and migration.^4^^,^^5^ Fibrillar collagens, such as type I, II, III, and V collagen, are synthesized as pro-α chains, named after their typical, large, left-handed α helical domain consisting of Gly-Xaa-Yaa repeats, with Xaa and Yaa often being proline and hydroxyproline, respectively.^6^^,^^7^ This large collagenous (triple helical) domain is flanked by smaller collagenous and non-collagenous (*i.e*., non-helical) domains, which make up the amino- (N-) and carboxy- (C-) terminal propeptides.^7^ During the maturation of the fibrillar procollagens, these propeptides are cleaved by specific N- and C-proteinases. Finally, the mature collagen molecules aggregate spontaneously into collagen fibrils.^7^, ^8^, ^9^, ^10^

**Figure 1:**
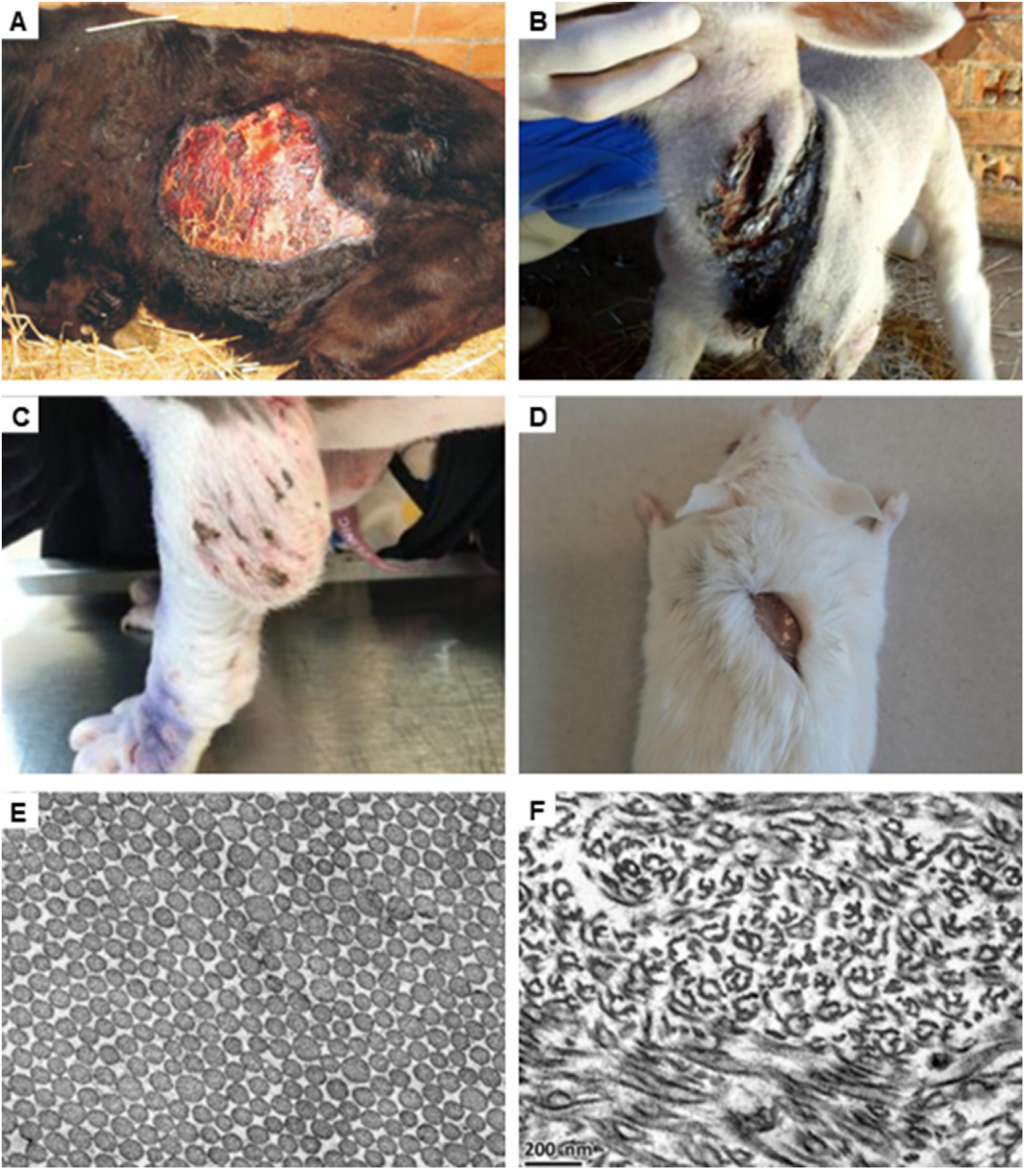
Clinical and ultrastructural manifestation of dermatosparaxis in different animal species. **(A)** A large and irregular tear in the skin of a male Drakensberger calf.^143^**(B)** Deep lacerations in the neck skin of a Dorper lamb.^144^**(C)** Joint swelling, atrophic scarring, and multifocal wounds on the front paw of an Alapaha blue blood bulldog.^145^**(D)** Skin tear in a transgenic *Adamts2*^*−/−*^ mouse caused by gentle scruffing (picture courtesy of Prof. Colige). **(E, F)** Transmission electron microscopy image of cross section of the dermal collagen fibrils of a wild-type Dachshund and a dermatosparactic Alapaha blue blood bulldog.^145^

In 1967, Hanset and Ansay reported that selective inbreeding in a Belgian cattle population had introduced an unwanted, autosomal recessive connective tissue disorder.^11^ Affected animals showed extreme skin fragility, resulting in avulsion of part(s) of the skin and subsequent sepsis, often leading to premature death. The condition was named dermatosparaxis (“tearing of skin”) and was later also reported in dogs, sheep, and cats (Fig. 1A–C).^12^, ^13^, ^14^, ^15^ Transmission electron microscopy analysis of the dermis of affected animals revealed a large proportion of disorganized, hieroglyph-like collagen fibrils (Fig. 1E, F). A substantial proportion of these malformed collagens contained two previously undescribed collagen chains that had a virtually identical amino acid (AA) composition to the α1 and α2 chains of mature type I collagen but carried N-terminal extensions resulting in a higher molecular weight.^16^ These N-terminal peptides caused steric hindrance and abnormal packing of the collagen fibers, leading to the observed “hieroglyphic” appearance, decreased collagen cross-linking, and decreased tensile strength, resulting in the fragile skin phenotype of the animals.^17^, ^18^, ^19^, ^20^ In 1960, Schmitt postulated that collagen polymerized in connective tissues following proteolytic conversion of soluble collagen precursors (*i.e.*, procollagen chains).^21^ Lenaers and Lapière confirmed his theory by showing that a novel enzyme, which they coined “procollagen peptidase” (or procollagen I N-proteinase, pNPI) was absent in the skin of dermatosparactic calves.^16^^,^^17^ They also showed that pNPI from healthy calf skin could cleave the aberrant pNα chains (α chains still containing the N-terminal propeptide) of dermatosparactic calf skin.^17^ They thus revealed for the first time that collagen molecules are produced as procollagens, requiring proteolytic maturation to achieve their biological function.^16^^,^^17^

## Structure and function of the procollagen peptidase ADAMTS2

**Figure 2:**
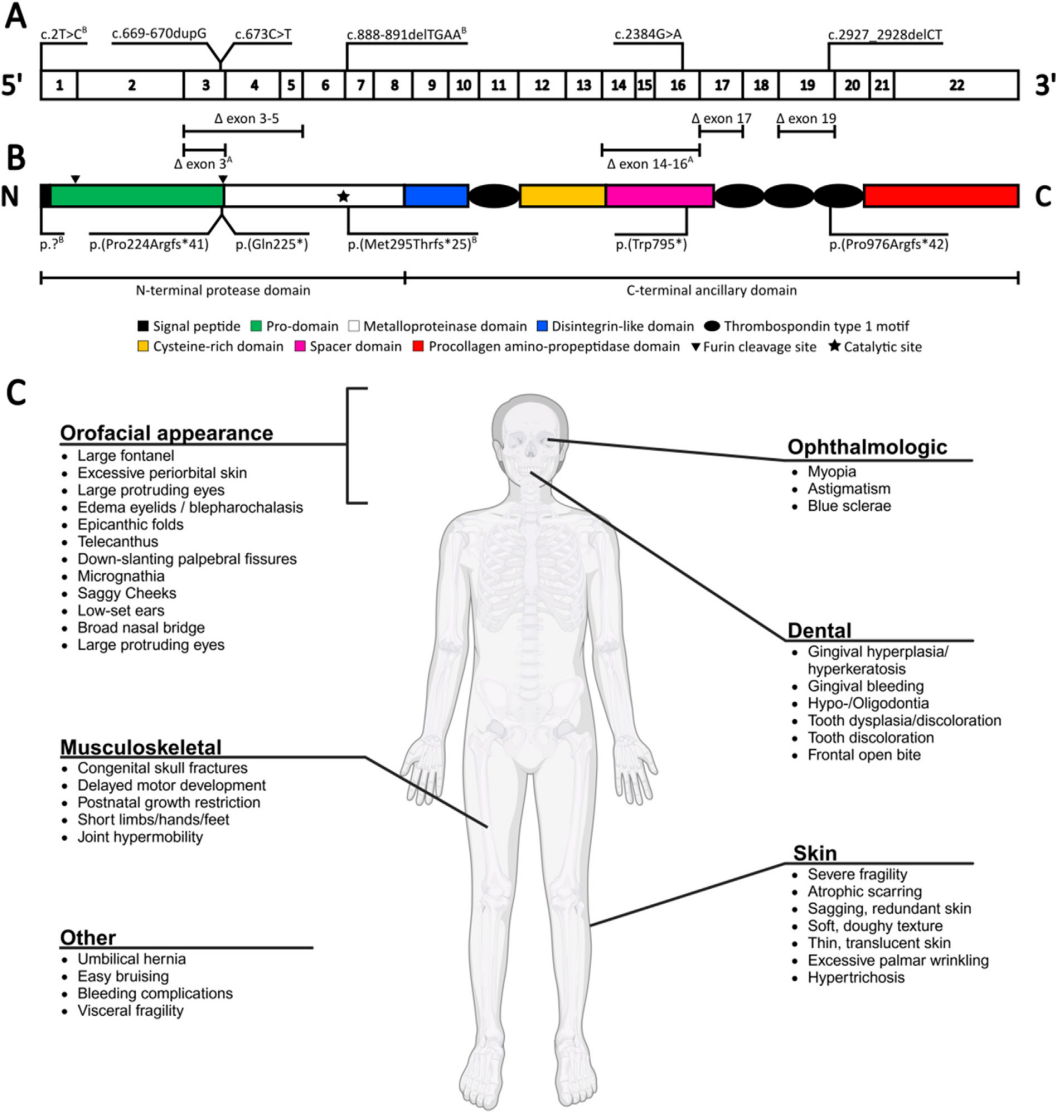
Schematic representation of the exon **(A)** and protein domain **(B)** structure of *ADAMTS2*. The ADAMTS2 protein consists of an N-terminal protease domain and a C-terminal ancillary domain.^24^ The N-terminal protease domain consists of a short signal peptide, a pro-domain, and the metalloproteinase domain, containing the catalytic site (star). The larger C-terminal domain comprises the disintegrin-like domain, a singular thrombospondin type 1 motif repeat (TSR) followed by a cysteine-rich domain, a spacer domain, and three more TSRs, ending with a C-terminal procollagen N-propeptidase domain. All known pathogenic variants causing Ehlers-Danlos syndrome dermatosparaxis type (dEDS) are indicated both at the gene (NM_014244.5) and protein level. Variants indicated with superscript A and B occur as compound heterozygous; all other variants are found in homozygous state. **(C)** Summary of the clinical features observed in dEDS made using biorender.com.

Colige and colleagues identified the procollagen peptidase as a disintegrin and metalloprotease with thrombospondin motif 2 (ADAMTS2), a 1211 AA-long enzyme encoded by the *ADAMTS2* gene (gene structure in Fig. 2A).^22^ This zinc metalloproteinase is part of the ADAMTS proteinase family,^23^, ^24^, ^25^, ^26^ which is evolutionarily and structurally related to the a disintegrin and metalloproteinase (ADAM) family, and more distantly to the matrix metalloproteinase (MMP) enzymes.^27^ They are part of the metzincin protease superfamily, sharing a characteristic conserved methionine residue close to their zinc ion-dependent active site.^27^ Within the ADAMTS family, ADAMTS2 is part of the procollagen N-propeptidases “clade”, together with ADAMTS3 and ADAMTS14. These enzymes share a highly similar AA sequence, domain organization, and function.^24^^,^^28^, ^29^, ^30^, ^31^, ^32^, ^33^

ADAMTS2 has two major domains, an N-terminal protease domain and a C-terminal ancillary domain that plays a role in substrate recognition and interactions with the cell surface (detailed domain structure in Fig. 2B).^24^ Multiple glycosylated sites lie within the C-terminal domain, but their biological function remains unknown.^24^ C-mannosylation and O-fucosylation of Trp and Ser/Thr, respectively, in the thrombospondin type 1 motif repeats (TSRs) of other ADAMTS enzymes has been reported, but there is no evidence of these modifications in ADAMTS2.^34^, ^35^, ^36^, ^37^ Initially synthesized as an inactive zymogen, proADAMTS2 is activated within the secretory pathway upon cleavage between its pro-domain and metalloproteinase domain by furin-like convertases (Fig. 2B).^24^ Although removal of the pro-domain is required for full enzymatic activity of ADAMTS2, recombinant enzymes lacking (part of) the pro-domain exhibit no enzymatic activity, indicating a potential role for the pro-domain in the correct folding of ADAMTS2. Following removal of the pro-domain, an autocatalytic cleavage partially removing the procollagen N-propeptidase domain results in a strong (3 to 4-fold) increase of enzymatic activity, suggesting a negative regulatory function for this domain. Finally, the second and fourth TSR are required for full enzyme activity, indicating a potential role in substrate recognition and/or binding.^24^ An alternative splicing mechanism resulting in a short form of ADAMTS2 consisting of the 543 N-terminal AA (exons 1–10) and 23 AA encoded by an alternative exon lying within intron 10 has been described.^23^^,^^26^ Although this short form contains the metalloproteinase domain, no detectable procollagen N-proteinase activity was found, and its physiological relevance remains unknown.

Mature ADAMTS2 is secreted into the ECM, where it remains in close proximity to the cell, probably through interactions with the second TSR domain.^24^ There, or intracellularly,^10^^,^^38^ ADAMTS2 carries out its primarily identified and best characterized function as a procollagen N-proteinase.

## *ADAMTS2* expression and regulation

High levels of ADAMTS2 mRNA expression and enzymatic activity were found in all type I collagen-rich tissues in fetal calf, *i.e.,* skin, tendons, bones, and aorta as well as in heart, liver, retina, and muscle, while kidney showed disproportionately low *ADAMTS2* expression, and brain and thymus showed only trace levels.^28^ *ADAMTS2* expression in humans was evaluated using RNA sequencing data in two studies, revealing high expression in the uterus, endometrium, gall bladder, placenta, spleen, lung, and small intestine.^39^^,^^40^

*In situ* hybridization of *Adamts2*, *Adamts3*, and *Adamts14* during mouse embryogenesis demonstrated distinct tissue-specific expression patterns with temporal differences.^41^ *Adamts3*, but not *Adamts2* or *Adamts14*, was expressed in cartilage (with *Col2a1*, encoding the pro-α1 chain of type II collagen) throughout development and in bone and musculotendinous tissues (with *Col1a1*, encoding the pro-α1 chain of type I collagen).^41^ This is in line with the 5-fold higher expression of *ADAMTS3* in cartilage^29^ and supports that ADAMTS3 is the physiologically relevant type II N-procollagenase in humans.^29^ In contrast, *Adamts2*, but not *Adamts3* or *Adamts14*, was expressed in many tissues throughout mouse embryogenesis, including the lung mesenchyme, palate, intestinal wall, peritoneum and mesentery, urinary bladder wall, aorta, and skin.^41^ Whereas low levels of *Adamts2* expression (and no expression of *Adamts3* or *Adamts14*) were found in embryonic skin, both *Adamts2* and *Adamts14* were detected in the dermis of two-week-old mice.^41^ Additionally, an increase in the expression of *Adamts2* was noticed in migrating multipolar neurons from E15 to E17 mouse embryos.^42^ These observations suggest that strict regulation of *ADAMTS2* expression is necessary during development and physiological processes.

In line with the findings in tissues, *ADAMTS2* is predominantly expressed by fibroblasts and mesenchymal cells,^23^, ^24^, ^25^, ^26^ but expression was also demonstrated in other cell types, including endothelial cells,^43^ macrophages,^44^ neonatal rat cardiomyocytes,^45^ and migrating multipolar neurons.^42^

**Figure 3:**
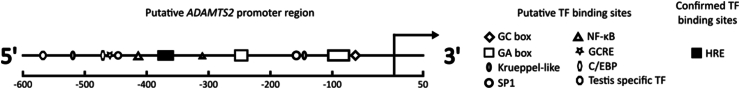
The putative *ADAMTS2* promotor region on which putative and confirmed transcription factor (TF) binding sites are indicated.

*In silico* studies of the genomic DNA upstream of *ADAMTS2* revealed a putative promoter region containing a GC box, two GAGA boxes, a CpG island, and several consensus recognition sequences of transcription factors known to regulate other ADAMTS genes (Fig. 3).^46^ *In vitro* transfection assays with deletion constructs of the putative *ADAMTS2* promoter driving luciferase expression revealed that the −658/+110 promoter region is sufficient for constitutive promoter activity, while the minimal −180/+110 bp region can induce lower levels of transcriptional activity.^46^

Several molecules that may induce *ADAMTS2* expression were identified in different *in vitro* cell lines and pathogenic conditions. Transforming growth factor beta 1 (TGFβ1), a well-known ECM formation activator, induces ADAMTS2 mRNA and protein levels in MG-63 cells (*i.e.*, human osteosarcoma cells of fibroblastic origin).^47^ Interleukin-1α and interleukin-6 induce ADAMTS2 mRNA and protein levels in MG-63 and Saos-2 cells, probably through Janus kinase (JNK)-mediated pathways.^46^^,^^48^ In human umbilical vein endothelial cells, hypoxia-inducible factor-1 (HIF-1) increases mRNA and protein levels by activating hypoxia response elements in the *ADAMTS2* promotor.^43^ In monocyte/macrophage cell lines, but not in epithelial/fibroblast lines or T and B lymphocytes, glucocorticoid treatment increases ADAMTS2 mRNA levels through interactions with the glucocorticoid receptor.^44^ Finally, in rat cardiac fibroblast cultures, MYC was recently shown to operate as a transcriptional activator of *Adamts2* expression.^49^

## Cleavage of the procollagen N-propeptide and dermatosparaxis phenotypes

As shown by the elegant studies on tissues from dermatosparactic animals and humans, ADAMTS2 is the principal type I procollagen N-proteinase.^28^ Overlapping expression between *Adamts2* and *Col3a1* (encoding the pro-α1 chain of type III collagen) and reduced pro-α1(III) processing in *Adamts2*^*−/−*^ mice, which likely contributes to loss of mechanical integrity of dermatosparactic skin, identified ADAMTS2 as the major type III procollagen-processing enzyme.^41^ A similar role in the maturation process of types II and V procollagen was subsequently reported *in vitro*.^24^^,^^47^ Initial studies revealed the potential preferential ADAMTS2 cleavage site between the small N-terminal collagen domain and the central collagen domains of the pro-α1(I), pro-α2(I), pro-α1(II), and pro-α1(III) chains.^26^ This site lies within a potential consensus cleavage sequence (AQESQAQ) and consists of a small aliphatic residue (P1) and a glutamine residue (P1'). In pNα1(V), cleavage occurs between a proline (P1) and alanine (P1') between its variable domain and short collagenous domain, although the consensus cleavage site is also present between its short (N-terminal) and main collagenous domain, indicating a second potential cleavage site.^24^^,^^26^ Recent N-terminomics studies revealed several ADAMTS2 cleavage sites in the N-terminal domain of the pro-α1(I), pro-α2(I), and pro-α1(V) chains, and one in the pro-α2(V) chain.^50^ This suggests that α1(V) and α2(V) chains may exist with N-terminal extremities of various sizes and bulkiness, which could be relevant for type V collagen-mediated regulation of collagen fibril formation.^50^

As mentioned, loss of the procollagen N-proteinase activity of ADAMTS2 in animals results in a disorder called dermatosparaxis.^16^^,^^17^ In 1992, a human counterpart for animal dermatosparaxis was identified in three infants.^51^^,^^52^ Affected children displayed a dermal phenotype characterized by extremely fragile skin and a typical “hieroglyphic” collagen fibril pattern on transmission electron microscopy images of skin. Sodium dodecyl sulfate polyacrylamide gel electrophoresis confirmed an accumulation of collagen chains with retained N-terminal propeptide in the skin, indicating a lack of the ADAMTS2 procollagen N-proteinase activity.^51^^,^^52^ Furthermore, when pNα chains extracted from dEDS patients were exogenously added to control cell cultures, they were converted into mature collagen molecules.^52^ The children were eventually diagnosed with “human dermatosparaxis”, now known as the dermatosparaxis type of EDS.^53^^,^^54^ To date, only 16 dEDS patients from 15 independent families were reported in literature,^23^^,^^51^^,^^52^^,^^55^, ^56^, ^57^, ^58^, ^59^ displaying a wide variety of clinical features, reviewed and summarized in Fig. 2C.^58^^,^^59^ In all patients, biallelic variants in the *ADAMTS2* gene were identified (Fig. 2B).^58^^,^^59^

A mouse model of dEDS was generated in 2001 by replacing exon 14 and parts of the flanking introns with a neomycin-resistance cassette using homologous recombination.^60^ These mice lack *Adamts2* expression, resulting in partially processed type I procollagen in their skin and other tissues.^60^ While normal at birth, homozygous *Adamts2* knockout (KO) mice (*Adamts2*^*−/−*^) develop recognizable differences compared with wild-type (WT) littermates within two months, with craniofacial abnormalities, thin, soft, and fragile skin (Fig. 1D), and decreased fur density with thinner hair follicles. Transmission electron microscopy images of skin revealed age-dependent differences between KO and WT littermates, with KO mice recapitulating the characteristic “hieroglyphic” collagen fibrils at approximately two months.^60^ A similar fragile skin phenotype was observed in two other *Adamts2*-KO lines, carrying a 28 and 245 bp deletion in the first exon of *Adamts2*.^61^ Lungs from two-week-old and two-month-old *Adamts2*^*−/−*^ mice had an emphysema-like appearance with consistently decreased parenchymal density.^41^ Significantly decreased type I and III procollagen processing was observed in these lungs, although histological analysis did not show collagen abnormalities.

An important differential diagnosis of dEDS is arthrochalasia EDS (aEDS), caused by heterozygous *COL1A1* or *COL1A2* defects leading to (partial) skipping of exon 6, which contains the ADAMTS2 cleavage site, causing retention of the N-propeptide of the affected collagen chain in mature collagen fibrils.^62^, ^63^, ^64^ Intriguingly, the phenotype of aEDS is quite different from that of dEDS.^53^ Whereas both the clinical and ultrastructural skin phenotype of aEDS patients is much milder compared to dEDS patients,^53^^,^^65^ aEDS patients have more outspoken generalized joint hypermobility, congenital bilateral hip dislocation, and multiple dislocations throughout life.^53^^,^^65^ The molecular basis for these phenotypic differences remains unknown, but the observation of a much more severe skin phenotype, and of dEDS symptoms that are not or to a lesser extent observed in aEDS patients, like bladder diverticula and cerebral aneurysm, suggests a major role for ADAMTS2 in the homeostasis of the affected tissues, possibly through cleaving other substrates. A mouse model for aEDS does not exist currently but would allow for an in-depth comparison with *Adamts2*^*−/−*^ mice.

## The roles of ADAMTS2 beyond its procollagen N-proteinase function

**Figure 4:**
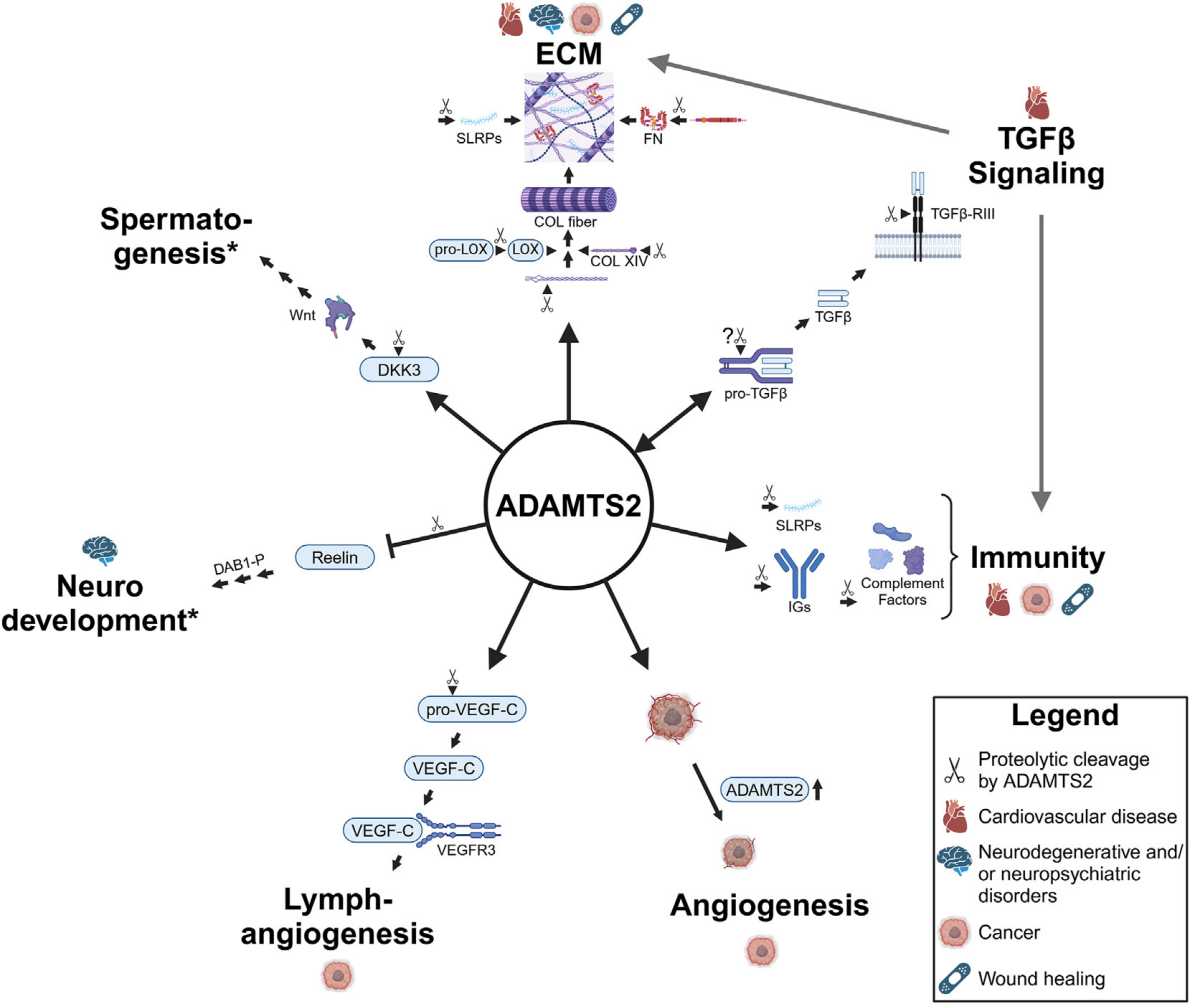
Overview of the different processes involving ADAMTS2 and their involvement in complex disorders. ADAMTS2 is involved in the organization of the extracellular matrix (ECM) through its role in the N-terminal maturation of several fibrillar collagens (type I, III and V) and lysyl oxidase (LOX) as well as through its potential substrates type XIV collagen (COL XIV), fibronectin (FN), and several small leucine-rich proteoglycans (SLRPs). It is potentially involved in transforming growth factor β (TGFβ) signaling through the proteolytic cleavage of pro-TGFβ and its receptor TGFβ-RIII. In turn, ADAMTS2 expression may be regulated by TGFβ itself. Multiple potential ADAMTS2 substrates, including the SLRPs, immunoglobulins (IGs), and several complement factors, are involved in immunity. Overexpression of ADAMTS2 in tumors implanted in nude mice results in smaller and less vascularized tumors, indicating a role for ADAMTS2 in angiogenesis, which is independent of its proteolytic activity. In lymphangiogenesis, ADAMTS2 is involved in the maturation of vascular endothelial growth factor C (VEGF-C). In neural development, ADAMTS2 can cleave and inhibit Reelin and subsequent phosphorylated Disabled-1 (Dab1-P) signaling. In spermatogenesis, ADAMTS2 cleaves Dickkopf-related protein 3 (DKK3), which in turn activates Wnt signaling. TGFβ signaling itself is important in ECM regulation and immunity, as indicated by two grey arrows. Furthermore, some potential ADAMTS2 substrates are involved in multiple processes, for example, some SLRPs are involved both in immunity and collagen fibrillogenesis, further enhancing the complexity of the role of ADAMTS2 in homeostasis and disorders. The asterisk (∗) indicates that next to neural development and spermatogenesis, Reelin and DKK3, respectively, are also involved in other processes, but these are not discussed here. This image was made using biorender.com.

N-terminomic proteome approaches and other experimental studies have been instrumental in revealing additional substrates of ADAMTS2 and/or have started to elucidate its multilevel regulatory role, either through its catalytic function or independent thereof (non-exhaustively listed in Table 1).^50^^,^^66^ These studies suggest that ADAMTS2 is, beyond its procollagen N-proteinase function, more broadly involved in the regulation of collagen fibril formation and function, but also in TGFβ signaling, angiogenesis, and lymphangiogenesis, neurodevelopment, spermatogenesis, and immunity (Fig. 4).

**Table 1:** Overview of reported ADAMTS2 substrates. A non-exhaustive list of different ADAMTS2 substrates is provided with the associated physiological process or signaling pathway, as well as the method that was used to obtain that information. Substrates with an asterisk (∗) were independently validated. Vimentin and actin can be cleaved by ADAMTS2, but it is not determined whether they are cleaved intracellularly or in the extracellular space after their release from dying cells.

Protein	Cleavage site	Evidence	Physiological process/pathway	Ref
**Collagens**				
Pro-α1(I) collagen chain∗	N-propeptide	N-TAILS (*in vitro*)	Collagen biosynthesis and fibrillogenesis and ECM organization	66
	Triple helix	N-TAILS (*in vivo*) *In vitro* digestion assay		50
	C-propeptide	N-TAILS (*in vivo*) *In vitro* digestion assay		50
Pro-α2(I) collagen chain∗	N-propeptide	N-TAILS (*in vitro*)	Collagen biosynthesis and fibrillogenesis and ECM organization	50,66
	Triple helix	N-TAILS (*in vivo*) *In vitro* digestion assay		50
	C-propeptide	N-TAILS (*in vivo*) *In vitro* digestion assay		50
Pro-α1(III) collagen chain∗	N-propeptide	*In vitro* digestion assay	Collagen biosynthesis and fibrillogenesis and ECM organization	41
	C-propeptide	N-TAILS (*in vitro*) *In vitro* digestion assay		66
Pro-α1(IV) collagen chain	^1438^G↓T^1439^ (C-terminal)	N-TAILS (*in vivo*)	Basement membrane	50
Pro-α1(V) collagen chain∗	N-propeptide & N-terminal	N-TAILS (*in vitro)*	Collagen fibrillogenesis	50
Pro-α2(V) collagen chain∗	N-propeptide & N-terminal	N-TAILS (*in vitro)* N-TAILS (*in vivo*)	Collagen fibrillogenesis	66 50
Pro-α1(VI) collagen chain	^186^F↓S^187^ (N-terminal)	N-TAILS (*in vivo*)	Basement membrane of skeletal muscle	50
Pro-α2(VI) collagen chain	^116^F↓S^117^ ^141^F↓A^142^ (N-terminal)	N-TAILS (*in vitro*) N-TAILS (*in vivo*)	Basement membrane of skeletal muscle	50,66
Pro-α3(VI) collagen chain	^1051^F↓A^1050^ (N-terminal)	N-TAILS (*in vitro*) N-TAILS (*in vivo*)	Basement membrane of skeletal muscle	50,66
Pro-α1(XIV) collagen chain	N-terminal (P630) C-terminal (P1636)	N-TAILS (*in vivo*)	Collagen fibrillogenesis	50
**Proteoglycans**				
Decorin		N-TAILS (*in vitro*)	Collagen fibril regulation Immune response control (elevates TLR response) TGFβ signaling	66
Biglycan		N-TAILS (*in vivo*)	Collagen fibril regulation Immune response control (upregulation B cell infiltration + elevates TLR response) TGFβ signaling	50
Lumican		N-TAILS (*in vivo*)	Collagen fibril regulation Immune response control (promoting Neutrophil chemotaxis) TGFβ signaling	50
Osteoglycin		N-TAILS (*in vivo*)	Collagen fibril regulation TGFβ signaling	50
**Glycoproteins**				
Fibronectin∗	N-terminal	N-TAILS (*in vitro*) *In vitro* digestion assay	ECM assembly	66
Reelin∗	N-terminal	*In vitro* digestion assay Western blot (mouse brain)	Neurodevelopment	61
**Collagen-modifying proteins**				
PCPE-1		N-TAILS (*in vivo)*	Fibrillar collagen maturation	50
Pro-lysyl oxidase	Central	*In vitro* digestion assay	Collagen fibril cross-linking	69
**Signaling molecules**				
TGFβ		N-TAILS (*in vitro*)	TGFβ signaling	66
LTBP1		Western blot (*in vitro* cell medium)	TGFβ signaling	66
TGFβ-RIII∗	Central (between endoglin-like and zona pellicuda domains)	N-TAILS (*in vitro*) Western blot (*in vitro* cell medium) *In vitro* digestion assay	TGFβ signaling	66
Pro-VEGF-C∗	N-propeptide	*In vitro* digestion assay Functional assay	Lymphangiogenesis VEGFR3 signaling	80
DKK3∗	^126^M↓V^127^ (N-terminal)	N-TAILS (*in vitro*) *In vitro* digestion assay	Testes development Organogenesis Carcinogenesis Wnt signaling TGFβ signaling	66
**Immune system**				
Immunoglobulins		N-TAILS (*in vivo*)	Immunity	50
Complement proteins (C3, C4-B, factor B, factor H)		N-TAILS (*in vivo*)	Immunity	50
Macrophage inhibitory factor		N-TAILS (*in vivo*)	Immunity	50
Annexins A8, A1 and A2		N-TAILS (*in vivo*)	Immunity	50
**Other**				
Actins∗	Several N- and C-terminal	N-TAILS (*in vivo*) *In vitro* digestion assay	Cytoskeleton	50
Vimentin∗	Several N-terminal and central	N-TAILS (*in vivo*) *In vitro* digestion assay	Cell integrity	50

Note: N-TAILS, amino terminal amine isotopic labeling of substrates; ECM, extracellular matrix; TLR, toll-like receptor; TGFβ, transforming growth factor β; PCPE-1, procollagen C-proteinase enhancer-1; LTBP1, latent TGFβ binding protein 1; TGFβ-RIII: TGFβ receptor III; VEGF-C, vascular endothelial growth factor C; DKK3, Dickkopf-related protein 3.

Of note, most substrates were found based on studies in dermal fibroblasts or murine skin, however, catalytic activity can be tissue-specific. In addition, not all the novel substrates are confirmed through independent techniques, and their biological and clinical relevance requires additional investigation, as these substrates can be important to understand the involvement of ADAMTS2 in physiological processes and diseases.

### A broader role of ADAMTS2 in ECM organization

Additional potential cleavage sites of ADAMTS2 were identified within fibrillar procollagen molecules and in other ECM (-related) molecules, including small leucine-rich proteoglycans (SLRPs), fibronectin, lysyl oxidase, and procollagen C-proteinase enhancer-1 (PCPE-1).

It was shown that ADAMTS2 could directly cleave and release the C-propeptide of the pro-α1 chain of type III collagen,^66^ and the presence of cleavage sites was also demonstrated within the C-propeptides of the pro-α1(I) and pro-α2(I) chains.^50^ Furthermore, multiple ADAMTS2 cleavage sites were found within the triple helical domain of the pro-α1(I) and pro-α2(I) chains. This could indicate a “collagenase” activity of ADAMTS2 potentially acting as a quality control mechanism, degrading incorrectly folded procollagen chains.^50^ In addition to the fibrillar collagens, ADAMTS2 can cleave the pro-α1, pro-α2, and pro-α3 chains of the beaded filament-forming type VI collagen.^50^^,^^66^ Potential cleavage sites were also found in the pro-α1 chain of type XIV collagen, a fibril-associated collagen with interrupted triple helices (FACIT) known for interacting with collagen fibrils and regulating their formation,^67^^,^^68^ and the pro-α1 chain type IV collagen.^50^ Aside from procollagens, potential proteolytic cleavage sites were identified in PCPE-1, a co-factor enhancing C-propeptide processing of fibrillar procollagens by bone morphogenic protein 1 (BMP-1),^50^ and in the N-terminal domain of both secreted and plasma forms of fibronectin,^66^ a glycoprotein required for collagen fibril assembly. ADAMTS2 was also shown to proteolytically process the lysyl oxidase precursor (pro-LOX), an enzyme essential for the formation of covalent cross-links stabilizing collagen fibrils.^69^ Furthermore, ADAMTS2 can process different proteoglycans regulating ECM assembly, including decorin, biglycan, lumican, and osteoglycin.^50^^,^^66^

### The role of ADAMTS2 in TGFβ signaling

The regulatory role of TGFβ1 on ADAMTS2 mRNA and protein levels in cell cultures has been mentioned above.^47^ However, ADAMTS2 is capable of modulating the TGFβ pathway. Bekhouche et al. identified many substrates within this pathway that can be cleaved by ADAMTS2, including latent TGFβ binding protein 1 (LTBP1), TGFβ-RIII, and probably pro-TGFβ, thereby indicating that ADAMTS2, -3, and -14 might play an important role in the regulation of TGFβ signaling.^66^

Furthermore, siRNA-mediated knockdown of ADAMTS2 in dermal fibroblasts resulted in an attenuated response of the TGFβ pathway upon stimulation with exogenous TGFβ1 and TGFβ2,^66^ suggesting that ADAMTS2 can process TGFβ regulators responsible for sequestering the TGFβ ligand in the ECM to reduce downstream effector activity. Together, these findings point to a positive feedback loop that could be involved in many processes.

TGFβ signaling plays a pivotal role in many physiological processes, including embryonic development, wound healing, tissue homeostasis (*e.g.*, through transcriptional regulation of ECM molecules), and immune homeostasis, and is also implicated in many pathophysiological processes such as developmental defects, aberrant healing, and fibrotic, inflammatory, infectious, and malignant disease.^70^ Hence, the effect of ADAMTS2 on TGFβ signaling could affect this wide range of (patho)physiological processes.

### The role of ADAMTS2 in angiogenesis

Angiogenesis is the process by which neovasculature is formed out of preexisting blood vessels. It is essential in normal development and wound healing, and plays a critical role in several pathological conditions, including cancer.^71^^,^^72^ Dubail and coworkers investigated the potential anti-angiogenic properties of ADAMTS2 and found that administration of recombinant ADAMTS2 to human umbilical vein endothelial cell and human dermal microvascular endothelial cell cultures led to inhibition of cell proliferation *in vitro*. The endothelial cells underwent morphological changes, resulting in loss of substrate adherence causing cell migration, establishment of new cell–cell contacts, and eventually cell cluster assembly, after which cells died probably due to anoikis-induced apoptosis.^73^ ADAMTS2 was immobilized by nucleolin at the endothelial cell surface, resulting in reduced extracellular signal-regulated kinase 1/2 (Erk1/2) phosphorylation, suggesting a role for Erk1/2 signaling in the disorganization of the actin cytoskeleton and causing changes in cell morphology. In embryoid bodies, vessel density was reduced following the addition of recombinant ADAMTS2, while overall growth was unaffected.^73^ *In vivo* choroidal neovascularization studies confirmed these findings as *Adamts2*^*−/−*^ mice displayed higher levels of neovascularization after sustaining laser burn injuries.^73^ Given the importance of angiogenesis in tumor growth and metastasis, Dubail et al. next showed that ADAMTS2-overexpressing HEK293-EBNA tumors engrafted in nude mice were smaller and less vascularized than control tumors. Interestingly, no accumulation of ADAMTS2 was found in the tumors, suggesting the death of the ADAMTS2-overexpressing cells, similar to the observations in endothelial cell cultures. These studies reveal a novel pro-apoptotic, anti-angiogenic, and anti-tumorigenic activity of ADAMTS2, which might be operated by one (or more) of its TSR domains, independently of its catalytic activity.^73^

### The role of ADAMTS2 in lymphangiogenesis

Vascular endothelial growth factor C (VEGF-C) and its interaction with VEGF receptor 3 (VEGFR3) are fundamental to normal lymphangiogenesis.^74^^,^^75^ Pro-VEGF-C is activated by cleavage of its N-propeptide by ADAMTS3 and its C-propeptide by furin and related proteins.^76^^,^^77^ *Adamts3-*deficient mice are unable to form a primary lymphatic network, causing embryonic lethality.^76^^,^^77^ In adult mice, *Adamts3* expression is restricted to cartilage and the central nervous system, but *Adamts2* and *Adamts14* are both more widely expressed. Recombinant ADAMTS2 and ADAMTS14 were both shown to be able to cleave pro-VEGF-C *in vitro*, resulting in VEGFR3 activation.^78^ *Adamts2*^*−/−*^ and *Adamts14*^*−/−*^ mice showed reduced lymphatic vessel diameter and spatial distribution in their dorsal ear skin, which was exacerbated in *Adamts2*^*−/−*^*;Adamts14*^*−/−*^ double KO mice. Lymphatic drainage of Evans blue dye, injected in mice footpads, was impaired in *Adamts2*^*−/−*^ and double KO mice, but not in *Adamts14*^*−/−*^ mice. Similarly, lymphatic density was reduced in the skin of 6-day-old *Adamts2*^*−/−*^ and double KO pups but not in *Adamts14*^*−/−*^ pups. Furthermore, *Adamts2*^*−/−*^ and double KO mice displayed lymphedema, but *Adamts14*^*−/−*^ mice did not. Finally, using thermal cauterization-induced corneal lymphangiogenesis as a model for lymphatic vessel neoformation in adults, Dupont et al. observed drastically reduced lymphangiogenesis in double KO mice, while *Adamts2*^*−/−*^ and *Adamts14*^*−/−*^ mice presented intermediate values suggesting equal involvement for both enzymes in this process.^78^ These data demonstrate that ADAMTS2 and ADAMTS14 are as efficient as ADAMTS3 for processing pro-VEGF-C into active VEGF-C and that their absence in mouse models *in vivo* leads to alterations of the lymphatic network in adulthood. However, considering that *Adamts2*^*−/−*^, *Adamts14*^*−/−*^, and double KO mice do not display massive lymphedema and that human dEDS patients have only mild signs of lymphatic dysfunction (*i.e.,* congenital edema of the eyelids in most, and generalized post-partum edema in one patient), their role in the generation of primary lymphedema seems overall limited.^58^ This potential role of ADAMTS2, -3, and -14 may be of importance in conditions where abnormal lymphangiogenesis is part of the pathology (*e.g.*, tumor aggressiveness), as a blockade of these enzymes could then be a potential therapeutic strategy in these conditions.

### The role of ADAMTS2 in neurodevelopment

Reelin is a large ECM glycoprotein that is associated with regulating neuronal migration in embryonic development and in adulthood and that promotes synaptic plasticity, cell adhesion, and cell proliferation. Most of our understanding of Reelin relates to the central nervous system, wherein Reelin has been considered a homeostatic regulator of synaptic plasticity.^79^ It is mainly expressed in Cajal-Retzius cells in the neocortex and hippocampus, in the granule cells in the cerebellum, and in other neurons in various regions, where it regulates the migration of differentiating neurons. In embryonic and early postnatal stages, Reelin activates a signaling cascade involving Disabled-1 (Dab1) phosphorylation, which plays a role in the neuronal lamination of the brain cortex, hippocampus, and cerebellum, as well as in the guidance of cortical neurons.^79^ Outside the central nervous system, Reelin is expressed in the liver, kidney, enteric nervous system, bone marrow, lymphatic tissues, and blood cells, serving a plethora of functions, including regulation of cell growth, cell cycle, cell motility, cell adhesion, hemostasis, and platelet spreading.^80^

A specific proteolytic cleavage of Reelin abolishes its biological activity, and ADAMTS3 was shown to be the major cleavage enzyme in the embryonic and early postnatal brain.^81^ Yamakage and colleagues, however, showed that ADAMTS2 could also cleave and thereby inactivate Reelin *in vitro.*^61^ *In vitro* inactivation of Reelin by recombinant ADAMTS2 and ADAMTS3, but not ADAMTS14, was shown to cause loss of Dab1 phosphorylation and decreased downstream signaling.^61^^,^^82^^,^^83^ Using *Adamts2*^*−*/−^ mice, ADAMTS2 was shown to significantly contribute to the N-terminal cleavage and inactivation of Reelin in the postnatal cerebral cortex and hippocampus, but much less in the cerebellum.

Reduced expression and signaling of Reelin has been reported with aging and in a number of neurological diseases, including ataxias,^84^ Alzheimer's disease,^85^ schizophrenia,^86^^,^^87^ autism,^88^ and even traumatic brain injuries.^89^ ADAMTS3 and ADAMTS2 may thus serve as potential therapeutic targets for these disorders.

### The role of ADAMTS2 in immunity

There is accumulating evidence that ADAMTS2 may significantly influence immune function. First, analysis of proteins cleaved by ADAMTS2 and ADAMTS14 in mouse skin revealed potential cleavage sites in several immune components, including immunoglobulins, complement proteins (C3, C4-B, factor B, and factor H), and factors involved in leukocyte activation. Potential substrates for ADAMTS2 include SLRPs (*e.g.*, biglycan, lumican, and osteoglycin), proteins that regulate collagen and play a role in immune responses.^44^ These findings point towards a broader role for ADAMTS2 and ADAMTS14 in immune regulation and suggest that further investigation is needed to confirm these findings and fully elucidate the mechanisms involved.

Secondly, a role for ADAMTS2 in the immune system was also suggested by the observation of increased expression levels of *ADAMTS2* mRNA and protein in monocytes and alveolar macrophages upon glucocorticoid treatment of *in vitro* peripheral blood mononuclear cells.^44^ Glucocorticoid treatment suppresses MMPs (*e.g.*, MMP-2 and -9), which are activated in macrophages during inflammation and hydrolyze several ECM proteins, including collagens.^90^, ^91^, ^92^ Surprisingly, this increase in *ADAMTS2* expression upon glucocorticoid treatment was not observed for cell lines of fibroblast or epithelial origin, suggesting that this *ADAMTS2* expression is cell-specific. This macrophage-specific increase of *ADAMTS2* expression might favor local collagen and ECM deposition in wound repair.^93^

The potential anti-inflammatory role for ADAMTS2 is further strengthened by the observation that *Adamts2*^*−/−*^*;Adamts14*^*−/−*^ mice develop epidermal lesions due to a dysregulated immune system.^94^ These lesions are preceded by an accumulation of immune cells (mainly T lymphocytes) in the dermis, seemingly inducing apoptosis of keratinocytes and local ruptures of the epidermis.^94^ Fluorescence-activated cell sorting analyses on blood samples of these mice indicated that the most consistent differences between *Adamts2*^*−/−*^*;Adamts14*^*−/−*^ mice and their WT controls were related to T lymphocytes, which were more activated and secreted higher levels of interleukin-2 (IL-2) and interferon-gamma (IFNγ). Similar trends were also observed for both cytokines in *Adamts14*^*−/−*^ mice and for IFNγ in *Adamts2*^*−/−*^ mice, even though these mice do not develop epidermal lesions. This observation suggests that the absence of these two enzymes has a direct global impact on the regulation of the immune system, not only in the skin.

Moreover, these findings strongly point to a role for ADAMTS2 in inflammation, wound repair, and tissue remodeling, which is both associated with and distinct from its collagen-related function.

### The role of ADAMTS2 in spermatogenesis

Male *Adamts2*^*−/−*^ mice were described to be sterile due to a lack of active mature sperm, and histological studies of testes revealed seminiferous tubules with reduced thickness and increased lumen in *Adamts2*^*−/−*^ mice.^84^ Interestingly, the secreted glycoprotein Dickkopf-related protein 3 (DKK3) was identified as a substrate of ADAMTS2.^66^ DKK3 acts as a modulator of Wnt signaling and TGFβ signaling during organogenesis and carcinogenesis^95^ and is involved in testis development.^96^ Hence, reduced DKK3 cleavage in *Adamts2*^*−/−*^ mice is suspected to inhibit Wnt signaling and could potentially influence seminiferous tubule development, resulting in the observed histological changes and male infertility.^84^^,^^85^ To our knowledge, human male dEDS patients have hitherto not procreated, and it remains to be determined whether this phenotype of male sterility is also observed in humans.^58^^,^^59^ Examination of patient-donated semen, evaluation of sperm numbers and morphology, and a testicular biopsy could provide further insights. Given the involvement of DKK3 in versatile functions, additional indirect consequences of its cleavage or lack thereof are possible.

## The complex role of ADAMTS2 in acquired disease

Recently, ADAMTS2 has been implicated in several complex acquired human conditions. These findings suggested novel functions unrelated to its catalytic activity and will be discussed below.

### The role of ADAMTS2 in the progression of cancer

In general, cancer cells are of epithelial origin and do not express ADAMTS2. Some cancer cells that acquire a mesenchymal phenotype (such as HS578T, an epithelial breast cancer cell line) do express significant amounts of ADAMTS2. A role for ADAMTS2 in cancer *in vivo* was first described in 2004 by Roemer et al.^97^ In the following years, ADAMTS2 was implicated in multiple cancer types.^97^, ^98^, ^99^, ^100^, ^101^, ^102^, ^103^, ^104^, ^105^, ^106^, ^107^, ^108^, ^109^, ^110^, ^111^, ^112^, ^113^, ^114^, ^115^, ^116^, ^117^ The available literature is summarized in Table 2. It is, however, important to note that several of the referenced articles use transcriptomic data extracted from total tumor tissues, and thus, *ADAMTS2* expression may correlate with desmoplastic reactions. In addition, proper ADAMTS2 detection could be hampered by potential problems concerning antibody specificity. Hence, these results need careful interpretation and validation. In many cancers, the presence of dysregulated ADAMTS2 expression was associated with worse outcomes, but mechanistic insight is generally lacking. The involvement of ADAMTS2 in cancer and cancer progression may be related to its aforementioned role in ECM organization, as ECM structure and composition changes are associated with tumor progression, and its role in lymphangiogenesis as the lymphatic system is indispensable for tumor growth and metastasis.^118^^,^^119^ Similarly, its postulated anti-inflammatory role may be involved in tumor progression, as tumors are known to employ a wide array of immune escape mechanisms to stop the immune system from being activated by tumor antigens.^120^ This contrasts with the anti-angiogenic and anti-tumoral properties of ADAMTS2 that were reported by Dubail et al. (discussed above)^73^ and the association of increased *ADAMTS2* expression and good prognosis in colorectal cancer.^100^

**Table 2:** Overview of different types of cancer ADAMTS2 is involved in, and detailing ADAMTS2 involvement and associated prognosis. It is important to note that several of the referenced articles use transcriptomic data extracted from total tumor tissues, thus, *ADAMTS2* expression may correlate with desmoplastic reactions. In addition, proper ADAMTS2 detection could be hampered by potential problems concerning antibody specificity. Hence, these results need careful interpretation and validation.

Type	Involvement of ADAMTS2	Prognosis	Reference
Colorectal cancer	*ADAMTS2* overexpression in CRC tissues (*n* = 4) vs healthy precancerous tissues (*n* = 2)	Not mentioned	98
	Altered *ADAMTS2* methylation in CRC samples vs peri-tumoral non-malignant tissue (*n* = 117)	Not mentioned	99
	Elevated ADAMTS2 levels in non-metastasized CRC tumors (*n* = 11) vs metastasized CRC tumors (*n* = 8).	Good prognosis	100
T/myeloid mixed phenotype acute leukemia	Complex rearrangement between chr 5 and 14 involving the ADAMTS2 locus resulting in upregulated *ADAMTS2* gene expression (*n* = 1). Acute lymphoblastic leukemia-based treatment achieved optimal response, normalizing *ADAMTS2* levels.	Unknown	101
Gastric cancer	*ADAMTS2* overexpression in GC tumor tissues (*n* = 171) vs healthy controls (*n* = 74) from 4 different datasets from the GEO database.	Not mentioned	102
	*ADAMTS2* overexpression in GC group vs healthy group (*n* = ?) in datasets from the TCGA and GTEx databases. *ADAMTS2* overexpression in paired GC tissues and adjacent non-cancerous tissues (*n* = 20).	Bad prognosis in men	103
	Elevated ADAMTS2 levels in cytoplasm of tumor cells and fibroblasts of gastric carcinoma vs noncancerous tissues (*n* = 655).	Bad prognosis	104
	*ADAMTS2* overexpression in GC tumor tissues (*n* = 111) vs healthy gastric tissues (*n* = 21) in datasets from the GEO database.	Not mentioned	105
	*ADAMTS2* overexpression in GAC tissues (*n* = 171) vs normal tissues (*n* = 77) in datasets from the GEO database. Elevated ADAMTS2 levels in GAC tissues vs adjacent healthy tissue (*n* = ?)	Bad prognosis	106
Oral cancer	*ADAMTS2* hypermethylation in buccal rinse cells from cancer-free heavy smokers vs cancer-free non-smokers.	Potentially positive (adaptive/protective response)	107
	*ADAMTS2* overexpression in OSCC tissues vs healthy tissues (*n* = ?) in datasets from the GEO database.	Not mentioned	108
	*ADAMTS2* overexpression in metastasized OSCC tumors (*n* = 11) in comparison to non-metastasized tumors (*n* = 8)	Not mentioned	109
Breast cancer	*ADAMTS2* was identified as a candidate prognostic gene	Not mentioned	110
Pancreatic cancer	*Adamts2* promotes pancreatic stellate cell differentiation into pro-invasive myofibroblast differentiation in *in vitro* murine pancreatic cancer cell lines, probably through altered TGFβ signaling	Not mentioned	111
	*ADAMTS2* is involved in cell migration and invasion in pancreatic cancer. *ADAMTS2* overexpression in pancreatic cancer through epigenetic demethylation by FTO in datasets from the GEO database.	Poor prognosis	112
Chondrosarcoma	*ADAMTS2* is upregulated in insulin-treated chondrosarcoma cells *in vitro* on day 11 as insulin may mediate effects on chondrosarcoma progression, indicating potential negative effect of diabetes mellitus therapy on chondrosarcoma.	Not mentioned	113
Papillary thyroid carcinoma	*ADAMTS2* overexpression in PTC tissues vs adjacent non-neoplastic tissues (*n* = 6) Note: no *ADAMTS2* overexpression in malignant vs paired healthy PTC tissues (*n* = 59) in TCGA datasets.	Poor prognosis	114
Thyroid cancer	*ADAMTS2* shows differential expression within different thyroid adenoma subclasses and different follicular thyroid tumors, identifying it as a potential differential, diagnostic marker for thyroid cancer.	Not mentioned	115
Ovarian cancer	*Adamts2* overexpression and elevated Adamts2 levels in cultures of highly metastatic murine OC cells vs other murine OC cell lines. Elevated ADAMTS2 levels in omental metastases than in primary OC sites	Bad prognosis	116
Renal cell carcinoma	*ADAMTS2* overexpression in ccRCC tumor tissues (*n* = 535) vs paracancerous tissues (*n* = 72). Increased *ADAMTS2* expression with tumor progression.	Not mentioned	117
	*ADAMTS2* overexpression in cancerous RCC tissue (starting from T2 or greater tumors) vs paracancerous healthy tissue (*n* = 27).	Bad prognosis	97

Notes: CRC, colorectal cancer; GC, gastric cancer; GEO, gene expression omnibus; TCGA, The Cancer Genome Atlas; GTEx: Genotype-Tissue Expression portal; GAC, gastric adenocarcinoma; OSCC, oral squamous cell carcinoma; TGFβ, transforming growth factor β; FTO, fat mass and obesity-associated protein; PTC, papillary thyroid carcinoma; OC, ovarian cancer; ccRCC, clear cell renal cell carcinoma.

How ADAMTS2 affects cancer progression thus appears to be type-, stage-, or organ-specific, and further research is required to elucidate the role of ADAMTS2 in different cancers. Evaluation of the potential substrates and processes that depend on ADAMTS2 is needed in each cancer type and stage to investigate exactly how ADAMTS2 influences tumor progression. In addition, *in silico* analysis using existing databases with (single-cell) RNA sequencing and proteomics may provide additional insights. siRNA-mediated knockdown of ADAMTS2 in specific tumor cell lines or *in situ* in specific animal models may also aid in the evaluation of its role in specific tumor progression.

### ADAMTS2 and cardiovascular disease

A limited number of studies have suggested a role for ADAMTS2 in cardiovascular disease, including pediatric stroke, cerebral aneurysm formation, acute myocardial infarction, heart failure, and cardiac hypertrophy. A genome-wide association study revealed an association between *ADAMTS2* (and *ADAMTS12*, *ADAMTS13*, and *ADAMTS17*) and pediatric stroke.^121^ In a subsequent study, an *ADAMTS2* variant was identified as a risk factor for cerebral aneurysm.^122^ Considering the involvement of ADAMTS2, -3, and -14 in procollagen maturation and the role of ADAMTS13 in von Willebrand factor processing, the expression of several ADAMTS enzymes was investigated in acute myocardial infarction.^123^ In culprit plaques, ADAMTS2 and type I procollagen, together with ADAMTS3 and -13, but not ADAMTS14, were shown to be up-regulated in endothelial cells and macrophages in injured fibrous caps (*i.e.*, a layer of fibrous connective tissue found in atherosclerotic plaques).

The mechanisms by which ADAMTS2 is involved in the pathogenesis of stroke, aneurysm, and acute myocardial infarction require further research but may involve alterations in collagen deposition, fibronectin cleavage (implicated in platelet thrombus formation), TGFβ signaling, and inflammatory responses.

In 2016, Wang and co-workers further investigated the role of ADAMTS2 in the heart. They observed up-regulated ADAMTS2 expression in failing human hearts, in aortic banding-induced hypertrophic mouse hearts, and in *in vitro* cultures of angiotensin II-treated neonatal rat cardiomyocyte.^45^ To explore the exact role of ADAMTS2 during cardiac hypertrophy, they investigated the hearts of *Adamts2*^*−/−*^ mice. Although at baseline, these mice did not display a cardiac phenotype, they showed exacerbated cardiac hypertrophy, increased cardiac dilatation, and cardiac dysfunction after aortic banding surgery.^45^ In addition, remarkable interstitial and perivascular fibrosis was seen in aortic-banding treated WT mice, but this fibrosis was more prominent in the *Adamts2*^*−/−*^ mice. This phenotype of cardiac hypertrophy, fibrosis, and dysfunction was blunted in transgenic mice with cardiac-specific overexpression of ADAMTS2. These findings suggest that the loss of ADAMTS2 exacerbates pressure overload-induced cardiac hypertrophy. Additional gain- and loss-of-function experiments in neonatal rat cardiomyocyte further corroborated the role of ADAMT2 as a negative regulator of cardiac hypertrophy, seemingly through down-regulating the phosphoinositide 3-kinase (PI3K)/protein kinase B (AKT) signaling pathway, a known key contributor to cardiac hypertrophy.^45^ ADAMTS2 was also mentioned as a cardioprotective factor in a study where ADAMTS16 was identified as a promotor of cardiac hypertrophy.^124^

Given the essential role of TGFβ signaling in the progression of cardiovascular disease,^125^ Huang and coworkers recently analyzed the expression pattern of TGFβ-related genes during the progress of heart failure and cardiac hypertrophy, using public single-cell RNA sequencing and transcriptome datasets of heart failure and hypertrophic cardiomyopathy.^49^ They observed high TGFβ activity in cardiac fibroblasts and endothelial cells during the progress of heart failure, but ADAMTS2 overexpression (transcriptionally up-regulated by MYC) could reverse this, seemingly through the PI3K/AKT and mitogen-activated protein kinase (MAPK) pathways.

Together, these observations suggest an essential protective role for ADAMTS2 in the progression of heart failure and cardiac hypertrophy and identify ADAMTS2 and its signaling pathways as potential therapeutic targets. As contradictory findings have been published, further studies are necessary. Rau et al. applied a co-expression network algorithm to perform a systems-level analysis of left ventricle transcriptomes of mice treated with isoproterenol to mimic catecholamine-driven cardiac hypertrophy.^126^ Their results suggest that ADAMTS2 plays a key role in modulating the expression of other heart failure-related genes (including natriuretic peptide A (*Nppa)* and natriuretic peptide B (*Nppb)*) in response to isoproterenol treatment. These authors confirmed their results with siRNA-mediated *Adamts2* knockdown in cardiac myocytes, which also led to reduced cardiomyocyte hypertrophy. Of note, DKK3, a known substrate of ADAMTS2, was shown to be strongly co-expressed with *ADAMTS2*. DKK3 has a known cardioprotective role.^127^, ^128^, ^129^ As such, ADAMTS2 could operate through the regulation of DKK3 cleavage.

### ADAMTS2 in neurodegenerative and neuropsychiatric disorders

A 2012 transcriptome study revealed a strong association between increased *ADAMTS2* expression and schizophrenia in blood from affected individuals.^130^ This association was confirmed in two independent drug studies, where *ADAMTS2* expression was reverted to “normal” levels in schizophrenia patients' peripheral blood mononuclear cells upon antipsychotic treatment targeting dopamine neurotransmission at the D1 and D2 receptors.^131^^,^^132^ Mechanistically, *ADAMTS2* expression was activated by dopaminergic signaling (D1-class receptors) and by cyclic adenosine monophosphate (cAMP)/cAMP response-binding protein (CREB) and MAPK/Erk signaling. Treatment with antipsychotic drugs and selective protein kinase A (PKA) and mitogen-activated protein kinase kinase (MEK) inhibitors abrogated D1-mediated activation of ADAMTS2 in neuronal-like cells. Thus, D1 receptors signaling towards CREB activation might participate in the onset and clinical responses to therapy in schizophrenia patients by controlling ADAMTS2 expression and activity. Amplified ADAMTS2 activity may potentially lead to Reelin inactivation, which has also been suggested to play a role in schizophrenia.^86^^,^^87^ Of note, a recent transcriptome study also identified *ADAMTS2* as the gene most associated with cognitive decline in a cohort of patients suffering from Alzheimer's disease, and this *ADAMTS2* expression was related to amyloid and tau accumulation.^133^^,^^134^ Currently, no clear mechanistic link is known between *ADAMTS2* expression and Alzheimer's disease pathology, but dysregulation of the ECM may affect the blood–brain barrier,^135^ which could be involved. Also, the inactivation of Reelin by ADAMTS2 may be a pathogenic process since reduced Reelin levels were linked with worse Alzheimer's disease pathology in animal models.^136^

## Conclusions and perspectives

Through the study of dermatosparaxis, the essential role of ADAMTS2 as type I procollagen N-proteinase has been extensively characterized. Yet, some questions regarding the pathomechanisms underlying this condition in animals and humans remain unanswered. In *Adamts2*^*−/−*^ mice and in human dEDS patients, the percentage of fully matured type I collagen varies from 20% to 30% in the skin to 80% in tendons and almost 100% in bone, which indicates the existence of (an)other enzyme(s) able to compensate for the absence of ADAMTS2 activity.^51^^,^^137^ Studies in *Adamts3*^*−/−*^ and *Adamts14*^*−/−*^ mice have shown that this cannot be attributed fully to the latter two enzymes.^77^^,^^94^ Meprin α and/or β, two metalloproteinases that can cleave the N- and C-propeptides of recombinant type I and III procollagen *in vitro*^138^^,^^139^ could potentially supplement the absence of ADAMTS2 activity in a time- and tissue-dependent manner. In human dEDS patients, depending on the pathogenic variant, residual catalytic activity, for example, due to the production of low amounts of truncated ADAMTS2 proteins, could also explain the presence of some fully matured type I collagen molecules. The phenotypic differences between dEDS and aEDS are intriguing and hint towards a broader role for ADAMTS2 in dEDS pathogenesis, such as through impaired cleavage of other substrates.

N-terminomic proteome and other experimental approaches have considerably expanded the substrate repertoire of ADAMTS2. In addition, the available mouse models have been instrumental in unraveling ADAMTS2 biology and shedding light on the spatial and temporal implication of ADAMTS2 in diverse homeostatic processes. More recent studies, including transcriptome and single-cell RNA sequencing analyses, as well as *in vitro* experimental setups, have unveiled an emerging role for ADAMTS2 in a number of complex disorders, such as cancer, cardiac hypertrophy, heart failure, and neurodegenerative disorders. The involvement of ADAMTS2 in these disorders is unsurprising, considering the complex interplay of several processes and (signaling) pathways in which ADAMTS2 is implicated (Fig. 4), including TGFβ signaling, Wnt signaling, VEGFR3 signaling, and Erk1/2 signaling. Furthermore, some of the identified ADAMTS2 substrates are associated with multiple processes (*e.g.*, some SLRPs are involved in both immunity and collagen fibrillogenesis), illustrating the variety and the intertwined mechanisms that can be regulated by ADAMTS2 activity. Moreover, hitherto unexplained and sometimes paradoxical findings in cancer and cardiovascular disease may be related to tissue-specific factors, including ECM composition and involved pathways. Although further experiments are needed to elucidate the specific role of ADAMTS2 in these complex disorders, its almost ubiquitous presence throughout the body makes it an attractive therapeutic target. To date, no specific ADAMTS2-blocking therapeutics exist, but research into other ADAMTS enzyme-blocking strategies, such as ADAMTS4, -5 and -13 can provide valuable insights.^140^, ^141^, ^142^ Administration of recombinant ADAMTS2 or therapeutically enhancing its expression may also be of interest in some disorders (*e.g.*, in dEDS, for anti-angiogenic purposes in cancer, or potentially in cardiovascular disease). Alternatively, approaches interfering with the identified signaling pathways might also provide a potential therapy. Finally, in several cancers, ADAMTS2 may be useful as a prognostic biomarker since it has been linked to better/worse prognosis.

Taken together, ADAMTS2 is involved in a broad array of processes. Future research will undoubtedly lead to a better understanding of the wide variety of pathways and pathological mechanisms in which ADAMTS2 plays a role (minor or major) and, in so doing, to the discovery of new therapeutic strategies for some of the disorders and conditions discussed in this review.

## CRediT authorship contribution statement

**Ruben Vanlerberghe:** Writing – review & editing, Writing – original draft, Visualization, Conceptualization. **Alain Colige:** Writing – review & editing, Supervision, Funding acquisition. **Anne-Marie Malfait:** Writing – review & editing, Supervision, Funding acquisition. **Delfien Syx:** Writing – review & editing, Writing – original draft, Supervision, Funding acquisition, Conceptualization. **Fransiska Malfait:** Writing – review & editing, Writing – original draft, Supervision, Funding acquisition, Conceptualization.

## Funding

This work was supported by the Research Foundation Flanders (FWO), Belgium (No. 1842318N, 3G041519 to F.M.; 12Q5920N to D.S.); Ghent University (No. GOA019-21); The Ehlers-Danlos Society (to D.S. and F.M.); the National Institutes of Health [National Institute of Arthritis and Musculoskeletal and Skin Diseases (NIAMS)] (USA) (No. R01AR060364, R01AR064251, P30AR079206 to A.M.M.; 1R21AR085242-01 to A.M.M., D.S., and F.M.). A.M.M. is supported by the Klaus E. Kuettner, PhD, Chair of Osteoarthritis Research. A.C. is a “Senior Research Associate” of the Fonds de la Recherche Scientifique - Fonds National de la Recherche Scientifique (FRS-FNRS, Belgium), and his work is supported by grants from the FRS-FNRS (No. FC96394, J.0034.24), the Fonds Spéciaux de la Recherche (Université de Liège), and the Fondation Hospitalo-Universitaire Léon Frédéricq (Université of Liège, Belgium).

## Conflict of interests

Anne-Marie Malfait is a consultant for Orion, Averitas, Eli Lilly, and Novartis.
